# Variable Admittance Control of a Hand Exoskeleton for Virtual Reality-Based Rehabilitation Tasks

**DOI:** 10.3389/fnbot.2021.789743

**Published:** 2022-01-12

**Authors:** Alberto Topini, William Sansom, Nicola Secciani, Lorenzo Bartalucci, Alessandro Ridolfi, Benedetto Allotta

**Affiliations:** Department of Industrial Engineering, University of Florence, Florence, Italy

**Keywords:** wearable robots, rehabilitation robotics, hand exoskeletons, variable admittance control, virtual reality

## Abstract

Robot-based rehabilitation is consolidated as a viable and efficient practice to speed up and improve the recovery of lost functions. Several studies highlight that patients are encouraged to undergo their therapies and feel more involved in the process when collaborating with a user-friendly robotic environment. Object manipulation is a crucial element of hand rehabilitation treatments; however, as a standalone process may result in being repetitive and unstimulating in the long run. In this view, robotic devices, like hand exoskeletons, do arise as an excellent tool to boost both therapy's outcome and patient participation, especially when paired with the advantages offered by interacting with virtual reality (VR). Indeed, virtual environments can simulate real-life manipulation tasks and real-time assign a score to the patient's performance, thus providing challenging exercises while promoting training with a reward-based system. Besides, they can be easily reconfigured to match the patient's needs by manipulating exercise intensity, e.g., Assistance-As-Needed (AAN) and the required tasks. Modern VR can also render interaction forces when paired to wearable devices to give the user some sort of proprioceptive force or tactile feedback. Motivated by these considerations, a Hand Exoskeleton System (HES) has been designed to be interfaced with a variable admittance control to achieve VR-based rehabilitation tasks. The exoskeleton assists the patient's movements according to force feedback and following a reference value calculated inside the VR. Whenever the patient grasps a virtual object, the HES provides the user with a force feedback sensation. In this paper, the virtual environment, developed within the Webots framework and rendering a HES digital-twin mapping and mimicking the actual HES motion, will be described in detail. Furthermore, the admittance control strategy, which continuously varies the control parameters to best render the force sensation and adapt to the user's motion intentions, will be investigated. The proposed approach has been tested on a single subject in the framework of a pilot study.

## 1. Introduction

Exoskeletons are a promising technology with a vast range of applications from the military to the industrial fields, from healthcare to injury prevention in physically stressful jobs. Besides, such devices are not only used to support the human body but can be exploited to drive an external robot in a primary-replicas fashion (Huang et al., 2018; Petrenko et al., 2019) or in imitation learning applications (Huang et al., 2019; Hua et al., 2021). In the last decades, an increasing number of exoskeletons have been designed for patients affected by motor dysfunctions or disabilities and applied in rehabilitation therapies, guided training, or assistance in everyday actions (Molteni et al., 2018; Shi et al., 2019; du Plessis et al., 2021). Robot-based therapy has in fact been proved to be effective and beneficial for both patients, reducing recovery time while increasing results, and therapists, who can exploit real-time monitoring to assess progress and tune the exercises accordingly (Lum et al., 2002; Staubli et al., 2009).

The excellent mobility, characterized by 27 Degrees of Freedom (DOFs), the small size, and the intensive use make the hand one of the most challenging body parts to support with an exoskeleton. Nevertheless, Hand Exoskeletons Systems (HESs) are widely investigated as the hand's primary and crucial role in human's quality of life, making them extremely valuable (du Plessis et al., 2021). Key components in developing HESs are the mechanical design and the implementation of a proper control system. The former concerns the process that shall guarantee a coherent motion with the wearer's body; the latter regards instead the management of the exoskeleton motion that shall match the user's intentions. In this paper, the attention will focus on the control strategy.

Commonly, control techniques involve a combination of a low-level controller, usually a PID or a model-based inverse dynamics controller, and a high-level one, e.g., adaptive control, sliding mode, impedance/admittance model, and AI-based strategies (Anam and Al-Jumaily, 2012). Impedance/admittance control is largely used in applications that involve Human-Robot Interaction (HRI) since it allows to shape the perceived robot's dynamic properties (i.e., inertia, damping, and stiffness) while interacting with the surrounding environment (Song et al., 2019). Indeed, it has been observed that, in order to perform complex actions, like walking or grasping an object, the human body not only exerts a force through the muscles but also changes the limbs' impedance to adapt to the interaction with the various kinds of objects. This very same idea has been successfully applied in many robotic applications to perform a fluid and safe HRI. A field of particular interest arose to be Robotics for Medicine and Healthcare where impedance/admittance control strategies have been widely investigated in robotic rehabilitation for upper and lower limbs (Keemink et al., 2018) or, more recently, for post-stroke hand (Sandison et al., 2020) and arm (Qian et al., 2021) therapy. Another relevant feature of this strategy is the possibility of controlling simultaneously both position and contact forces in all the robot's workspace, thus keeping the interactions smooth and safe for the people, environment, and robot. This latter capability also sets apart admittance control from other hybrid position/force control strategies that divide the workspace into sub-regions.

Hand-in-hand with Robotics, another fast-growing technology is virtual reality (VR) with applications that span from education (Kavanagh et al., 2017; Radianti et al., 2020) and tourism (Yung and Khoo-Lattimore, 2019) to engineering design (Wang et al., 2018; Wolfartsberger, 2019) and surgical training (Pfandler et al., 2017; Bielsa, 2021). VR is low cost, has high flexibility, and great adaptability; these characteristics make it a noteworthy tool for rehabilitation allowing the design of personalized and safe sets of exercises and, at the same time, providing real-time feedback both for patients and therapists (Rose et al., 2018). Repetitive and boring sessions may become more stimulating, making the patient feel actively involved. Indeed, it has been demonstrated that VR training is an effective rehabilitation tool providing both short and long term improvement on motor functions and better psychological effects on patients over other traditional methods (de Araújo et al., 2019; Lei et al., 2019). Modern computers supply enough computational power that, combined with advanced developing tools and solvers, allow for the simulation of complex environments providing a very immersive experience.

Combining together exoskeletons with virtual environments, enhancing their inherent properties could lead to devices with an extraordinary capability to customize exercises and therapies. Additionally, given their flexibility, many applications can also be developed in different fields (e.g., pilot training).

### 1.1. Contribution and Paper Structure

This work's main contributions can be summarized in the following points:

- Development and validation of a VR environment for a HES comparing three different simulators: Gazebo^1^ Pybullet^2^ and Webots^3^.
- Study, implementation, and testing of two different admittance control strategies with parameter tuning on a custom-developed digital twin and refinement over a real exoskeleton.
- Link, through a robot operating system (ROS)^4^ architecture, the real exoskeleton to the virtual environment, thus enabling a user to physically perceive virtual objects on his hand through force feedback.
- Testing the whole system to assess its performance by means of a pilot study involving a single subject.

In this section, an outline of the motivation for the proposed work has been given, along with a first overview of the technical and theoretical tools employed. The remainder of the paper will be organized as follows: (i) Section 2 provides background information about the hand exoskeleton at the core of this work, the involved HRI framework, and the theoretical basis of the admittance control technique; (ii) Section 3 explores in detail about the whole design process, from the building and validation of the virtual environment to the description of the two admittance control strategies, through the explanation of each of the main design choices; (iii) Section 3 describes the design process from the beginning to the control strategy implementation; (iv) Section 4 outlines the achieved results at the end of a two-stage experimental test setup; (v) Section 5 concludes the paper exposing some final considerations.

## 2. Background

This section will present an overview of the hand exoskeleton exploited in this work, then the general framework for the HRI involved in this study, and, last, some background theory for admittance control. These topics reported here are useful background concepts for a comprehensive understanding of the subsequent sections.

### 2.1. The BMIFOCUS HES

The BMIFOCUS hand exoskeleton has been designed by a research team from the Mechatronics and Dynamic Modeling Laboratory (MDM Lab) at the Department of Industrial Engineering of the University of Florence (UNIFI DIEF) and MOV'IT S.r.l. (Pisa, Italy) as an innovative HES for Assistance-As-Needed (AAN) rehabilitation for tasks, such as grasping and pinching (Bartalucci et al., 2020). The previous exoskeleton already addressed the issue of mechanically reproducing complex finger kinematics with great accuracy exploiting a single-DOF rigid kinematism (Conti et al., 2017). This innovative device has been realized in the framework of the BMIFOCUS research project (funded by the Tuscany Region, Italy) on the basis of a previously developed prototype (Bartalucci et al., 2020). The HES has been redesigned to satisfy the new project requirements:

- the independent motion of, at least, three fingers (i.e., thumb, index, and middle finger);
- maximum load on each finger mechanism end-effector^5^: 20 N;
- reversibility for patients' safety in case of involuntary muscle contractions;
- the total mass of the wearable part below 0.5 kg;
- adaptability to different hand sizes.

**Figure 1:**
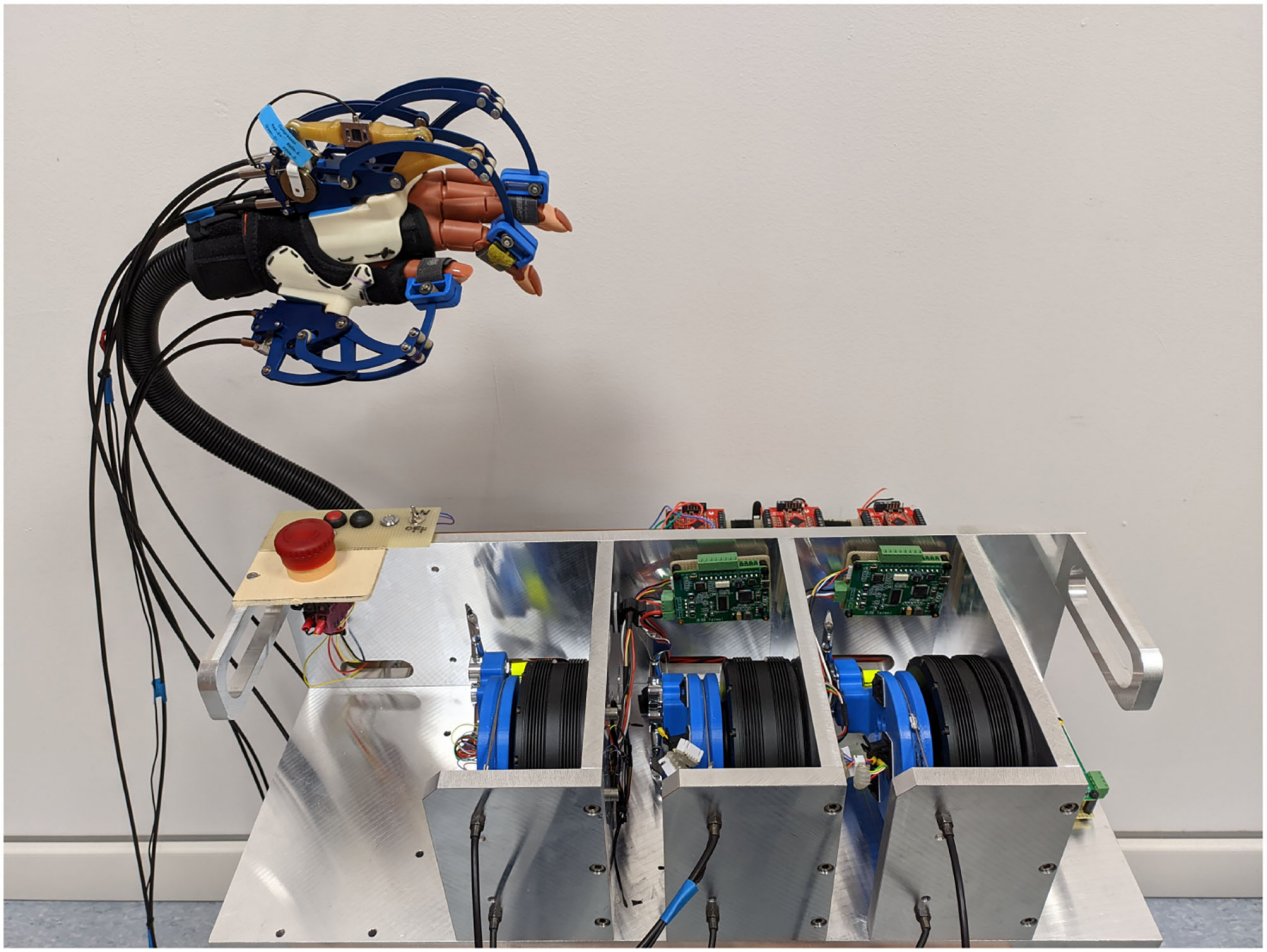
The hand exoskeleton system (HES) developed, designed and realized by the Department of Industrial Engineering of the University of Florence (UNIFI DIEF) within the Brain machine interface in space manned missions: amplifying FOCUSed attention for error counterbalancing research project. The figure shows the wearable part mounted on a mannequin hand and the remote actuation system in foreground.

**Figure 2:**
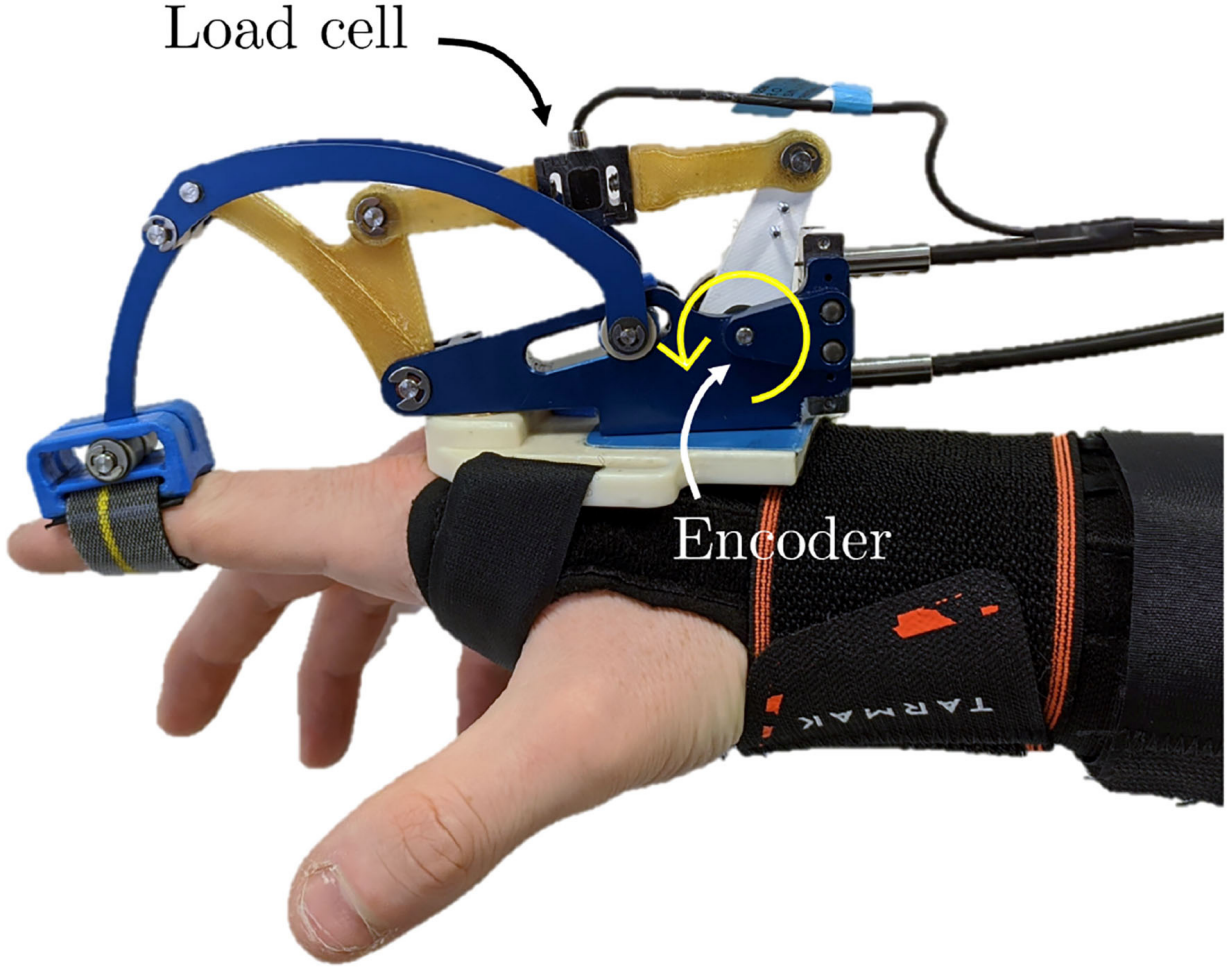
Overview of the sensors mounted on each finger mechanism. The yellow arrow identifies the only Degrees of Freedom (DOF) of the particular kinematic structure.

The BMIFOCUS HES is comprised of two distinct parts: the Remote Actuation System (RAS) and the wearable exoskeleton, shown in Figure 1, respectively, on the left and on the right. The wearable part is composed of a base platform housing from one to four different finger mechanisms made of aluminum alloy that exploit a four-bar linkage to actuate the finger. Encoders and load cells (one per each finger mechanism) provide feedback measurements of angular position, speed, and exerted force (as shown in Figure 2). The RAS has a modular structure to make each finger independent from one another. It is based on a Bowden-cable transmission system connected to each of the finger mechanisms by means of a custom pulley. Thanks to this structure, the system minimizes the number of components on the user's limb and allows for remote placement of the actuation system without limiting the user's movements. The actuation is performed by means of brush-less DC motors (one per each finger involved) speed-controlled by an independent PID controller specifically tuned for the corresponding finger. By design choice, the motors have no extra gears and the pulleys on the finger mechanisms have diameter four times smaller than the one of the motor pulleys: this guarantees that the user is always able to overcome the motor torque to avoid injuries during, for example, an involuntary muscle spasm.

### 2.2. Physical Human-Robot Interaction (pHRI)

A valuable framework for understanding pHRI is described in Losey et al. (2018), which provides guidance in designing such systems and setting proper requirements. The study identifies three crucial points in applications with shared control between humans and robots: intent detection, arbitration, and feedback.

**Intent detection** is defined as “*the need for the robot to have knowledge of some aspect of the human's planned action in order for the robot to appropriately assist toward achieving that action*.” This means that the control system needs to acquire some kind of signal and from this data infer the user's intention in order to properly drive the robot. Many possible approaches can be adopted. Complex data like ElectroEncephaloGraphy (EEG) or ElectroMyoGraphy (EMG) can be exploited to collect signals and then interpret them through machine learning algorithms, e.g., Support Vector Machines (SVMs), Hidden Markov Models (HMMs), or Artificial Neural Networks (ANNs). Conversely, intents can be deduced from simpler signals, like force or torque measurements, exploiting Kalman filters or other heuristics methods.

In this project, force measurements are acquired from load cells directly attached to the exoskeleton finger mechanisms. These measurements, as detailed in Section 3.2, have been either directly passed as a reference for the control system for the classical admittance control, or compared with the finger angular speed direction for the variable admittance control. In the latter case, the finger angular speeds, acquired from the magnetic encoders mounted on each finger mechanism, become part of the data necessary for intent detection.

**Arbitration** is intended as “*the division of control among agents when attempting to accomplish some tasks”* where the word *agents* refer to both human operator(s) and robot(s). Four types of arbitration can be distinguished: (i) *co-activity* where each agent performs his/its own sub-tasks; (ii) *primary-replica* where one agent (usually the robot) follows the other's intention (commonly the human operator); (iii) *teacher-student*, often referred to as AAN in rehabilitation, consists of “*attempting to train humans using robotic platforms”*; (iv) *collaboration* where human and robot work together to reach a desired goal.

The admittance control strategy adopted in our system performs a combination of the primary-replica and teacher-student kind of arbitration.

**Feedback** to the human operator can be provided through visual, aural, or force signals. It is easy to provide visual and aural information through monitors and speakers. Force feedback instead is more challenging and, at the same time, of great interest in HRI because of its similarity with sensors embedded in our muscles and skin. The most common wearable force feedback devices are based on vibration, skin stretch, or pressure while other technologies rely on direct nerve stimulation.

Losey et al. (2018) identify several benefits in combining visual and force feedback. In the proposed work, both have been used. Visual feedback is provided by means of VR environment representation on a computer screen, while force feedback is obtained from the exoskeleton's finger mechanisms acting on the user's fingers thus providing a way for the user to feel reaction forces computed in the VR when interacting with virtual objects.

**Figure 3:**
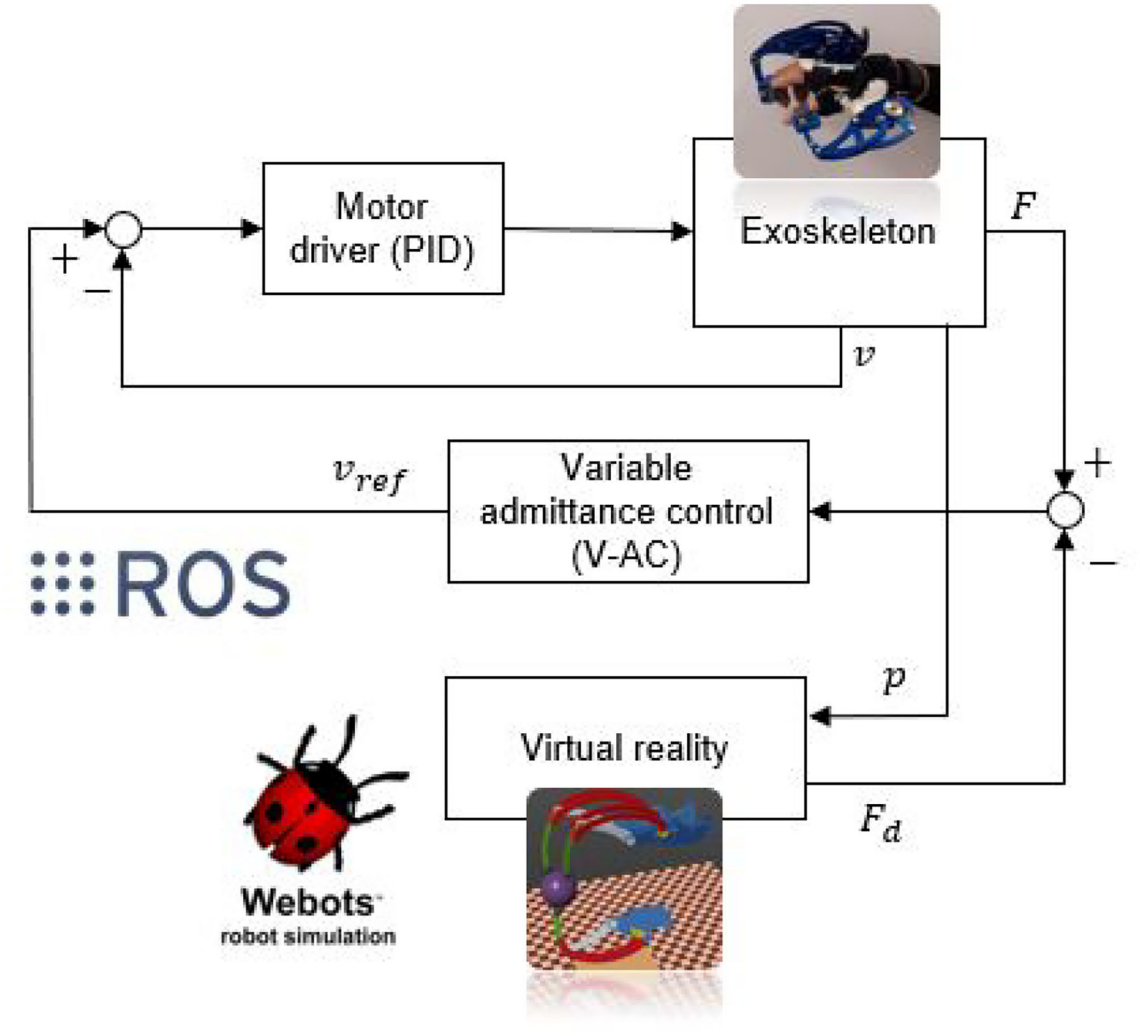
Overall control architecture of the proposed strategy. *F*, *v*, and *p* represent, respectively, the measured force, the angular speed, and the angular position of each finger mechanism; *F*_*d*_ highlights the high-level reference force computed within the VR; *v*_*ref*_ outlines the low-level reference speed quantified by the proposed admittance control.

Focusing on the proposed research activity, the overall pHRI architecture is reported in Figure 3. Intention detection is first performed exploiting the force sensors on the exoskeleton; specifically, the intention detection algorithms investigates if the user wants to accelerate or decelerate the motion of each finger independently. At the same time, the position sensors mounted on each of the finger mechanisms drive the motion of the virtual replica of the exoskeleton. While VR gives visual feedback to the user, the virtual reality controller calculates the possible interaction forces with virtual objects. This information is fed to the admittance control algorithm (high-level control) that provides the speed reference for each of the motors. PID-based motor drivers (low-level control) then track such references, allowing the exoskeleton to produce the desired motion.

In other words, the exoskeleton assists the patient's movements according to the detected intention and following a reference force value calculated inside the VR (which renders the digital-twin mapping and mimicking the real exoskeleton motion). Whenever the patient grasps a virtual object, the VR changes the reference force value and the HES provides the user with force feedback as he/she was physically interacting with it.

### 2.3. Impedance/Admittance Control

As already reported, impedance/admittance control is one of the most used strategies in exoskeletons ad rehabilitation robots (Anam and Al-Jumaily, 2012; Song et al., 2019). Its core idea is applying some corrections to the robot's trajectory in order to achieve a desired dynamic interaction between robot and environment. This is performed through two nested control loops: the high-level one that computes the desired dynamical behavior generating references for the low-level one that usually controls either the robot's position, force, or torque.

**Figure 4:**
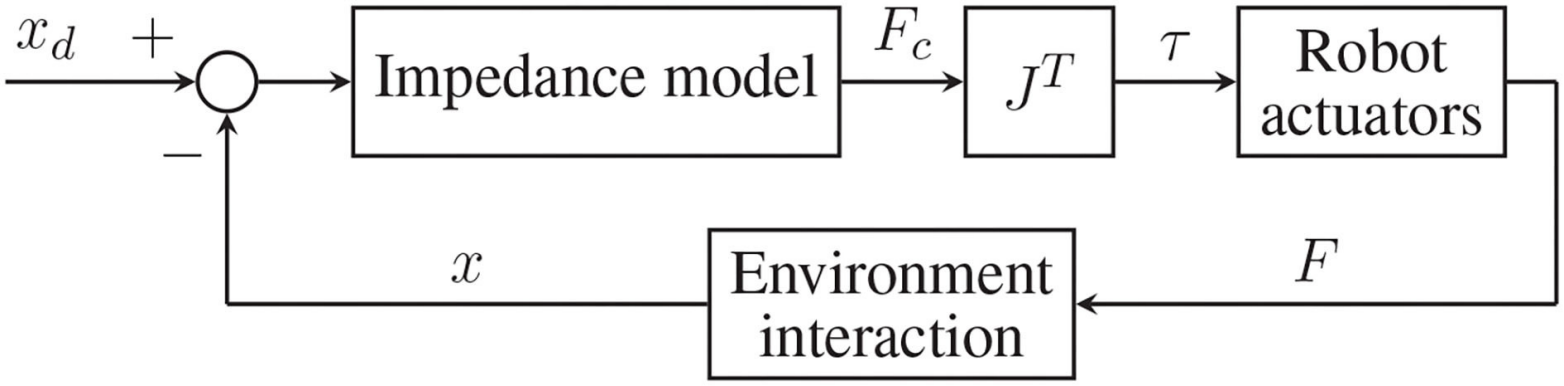
General scheme of an impedance (torque-based) control.

The *impedance control* technique, also known as force/torque-based control, exploits an impedance model that, starting from the error between the desired end-effector's position (*x*_*d*_) and the measured one (*x*), computes the desired contact force (*Fc*) between the robot and the environment (in this case, the human operator). An inner loop applies this torque reference (τ) to the robot actuators once mapped according to its transposed Jacobian (*J*^*T*^). The actual force (*F*) exerted by the robot on the environment then produce the actual end-effector's position (*x*), from which the further iteration starts. This control strategy scheme is shown in Figure 4.

**Figure 5:**
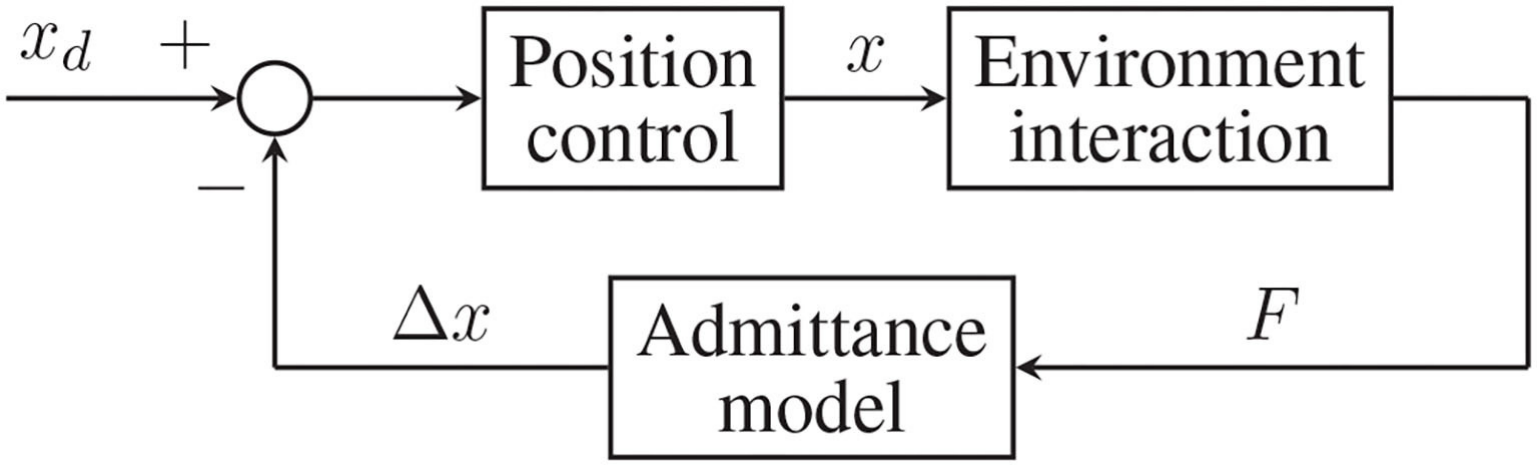
General scheme of the admittance (position-based) control.

The *admittance control* method, known instead as position-based control, adopts the opposite approach. First, the contact force (*F*) is measured and input into the admittance model that calculates a relative displacement (Δ*x*), intended as the estimated difference between where the robot is (*x*) and where it should be. Last, the error between the desired trajectory (*x*_*d*_) and the relative displacement guides the robot through a position control loop. This second implementation is shown in Figure 5.

Impedance and admittance control strategies are two sides of the same coin, both methods have their advantages and disadvantages, choosing one over the other depends on the kind of sensors present on the manipulator, whether the environment is stiff or soft, if it is more important to control precisely position or contact force in the given application, and other factors. Some guidelines are provided in Song et al. (2019) and Schumacher et al. (2019).

In the proposed study, the admittance control method has been preferred over the impedance control technique. The reason for this choice lays in the architecture of the system: according to Figure 3, the control strategy takes as input one or more force signals and calculates a reference speed value for the motors. The general control scheme, therefore, becomes of the position-based type, as the one shown in Figure 5.

#### 2.3.1. Mathematical Formulation

Mechanical impedance represents the relationship between motion and applied force (admittance is instead defined as the inverse of impedance), in Laplace domain, it is defined as:


(1) $$\begin{array}{l} Z\left(s\right)=\frac{F\left(s\right)}{\dot{X_{r}}\left(s\right)} \end{array}$$

where *X*_*r*_(*s*) is the relative displacement between actual and equilibrium position, i.e. *X*_*r*_(*s*) = *X*(*s*) − *X*_*d*_(*s*), *F*(*s*) is the applied force, and *Z*(*s*) is the impedance model, usually assumed in the following linear form Song et al. (2019):


(2) $$\begin{array}{l} Z\left(s\right)=Ms+B+\frac{K}{s} \end{array}$$

*M*, *B*, and *K* represent, respectively, the inertia, damping, and stiffness matrices and represent the model's parameters to be defined. By merging Equation 1 and 2, and translating the result into the time domain, the following is obtained:


(3) $$\begin{array}{l} M\left(\ddot{x}-{\ddot{x}}_{d}\right)+B\left(\dot{x}-{\dot{x}}_{d}\right)+K\left(x-x_{d}\right)=F\left(t\right) \end{array}$$

where *F*(*t*) represents the contact force, *x*(*t*) and *x*_*d*_(*t*) represent the actual and desired end-effector's position, respectively.

**Figure 6:**
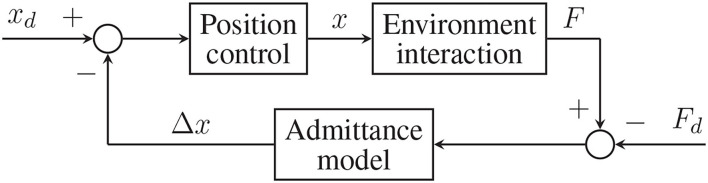
Force-tracking admittance control general scheme.

As a means to overcome some practical challenges in impedance control, like unknown environment's characteristics, a modified version of Equation 3 has been proposed in Seraji and Colbaugh (1997), Jung et al. (2004), and Roveda et al. (2015):


(4) $$\begin{array}{l} M\left(\ddot{x}-{\ddot{x}}_{d}\right)+B\left(\dot{x}-{\dot{x}}_{d}\right)+K\left(x-x_{d}\right)=F\left(t\right)-F_{d}\left(t\right) \end{array}$$

where *F*_*d*_(*t*) is the desired contact force. This strategy is named *force-tracking admittance control* and is represented in Figure 6.

Its advantage lies in the capability of following simultaneously a force and position reference while enforcing the motion characteristics (*K, B, M*) defined in the model. This property has been exploited in this project to provide force feedback computed within a virtual environment to a patient's hand wearing the exoskeleton (see section 4). Besides, the same feature could be used for AAN treatments if a proper fitting reference *F*_*d*_ is generated.

#### 2.3.2. Stability

Investigating the so-called *coupled stability* is considered one of the most efficient ways to study the stability of admittance control strategies (Song et al., 2019). This property consists in the stability for the robot-environment coupled system and it is crucial in our analysis because of the complex interactions that may occur. Additionally, two different operating modes are usually necessary, namely, the constrained and free motion, that is when the manipulator and environment are or are not in direct contact. In the free motion phase, the controller characteristics alone are enough to determine the system's stability, while during the constrained phase performance is influenced by the environment's properties.

A passivity criterion has been proposed for passive environments (Colgate and Hogan, 1988; Hogan and Buerger, 2018) to analyze coupled stability. However, in HRI, the “environment” is typically a limb that can hardly be modeled as passive since it can move through muscles activation. Therefore, the Passivity criterion should not be directly applied to such applications. Nonetheless, as reported in Kim et al. (2018), it has been proved that stability is preserved when the environment's stiffness is not too high, as in the case of human limbs.

In the proposed work, the trial and error process suggested in Lecours et al. (2012) has been used to identify the stability limit values for inertia and damping of the coupled system. The rigid component of the system *K* has been neglected by considering, as a first approximation, the limb interaction fully compliant with the exoskeleton motion. Such boundaries have been set by incrementally varying the parameter values until instability (shown as critical fluctuating vibrations) arose for minimum inertia of 0.005 *kgm*^2^ (*M* ≥ 0.005) and maximum damping of 0.25 *Ns*/*m* (*B* ≤ 0.25). The stiffness component of the system K has been neglected by considering, as a first approximation, the limb interaction fully compliant with the exoskeleton motion.

### 2.4. Advanced Implementations

Several new approaches derived from the classical admittance control described above have been proposed in the scientific literature (Ikeura et al., 2002; Sado et al., 2014; Li et al., 2017; Souzanchi-K et al., 2017; Song et al., 2019). Some of them are based on robust and adaptive methods, machine learning techniques, and variable impedance/admittance.

Robust impedance control has the purpose of maintaining desired mechanical dynamics in presence of model parametric uncertainties, unknown environments, and other common sources of disturbances. Solutions based on the sliding mode control technique have been proposed (Lu and Goldenberg, 1995). Other methods use neural networks to model uncertainty compensation (Jung and Hsia, 1998) or direct and indirect adaptive algorithms for online parameter modulation (Hogan, 1984; Tsumugiwa et al., 2002).

Learning techniques have been successfully employed to determine optimal impedance values and trajectories. Frequently used models are neural networks combined with reinforcement learning methods, these strategies are called “inverse dynamic model learning” or “nonlinear regulator learning” (Gomi and Kawato, 1993; Li et al., 2017; Song et al., 2019).

**Figure 7:**
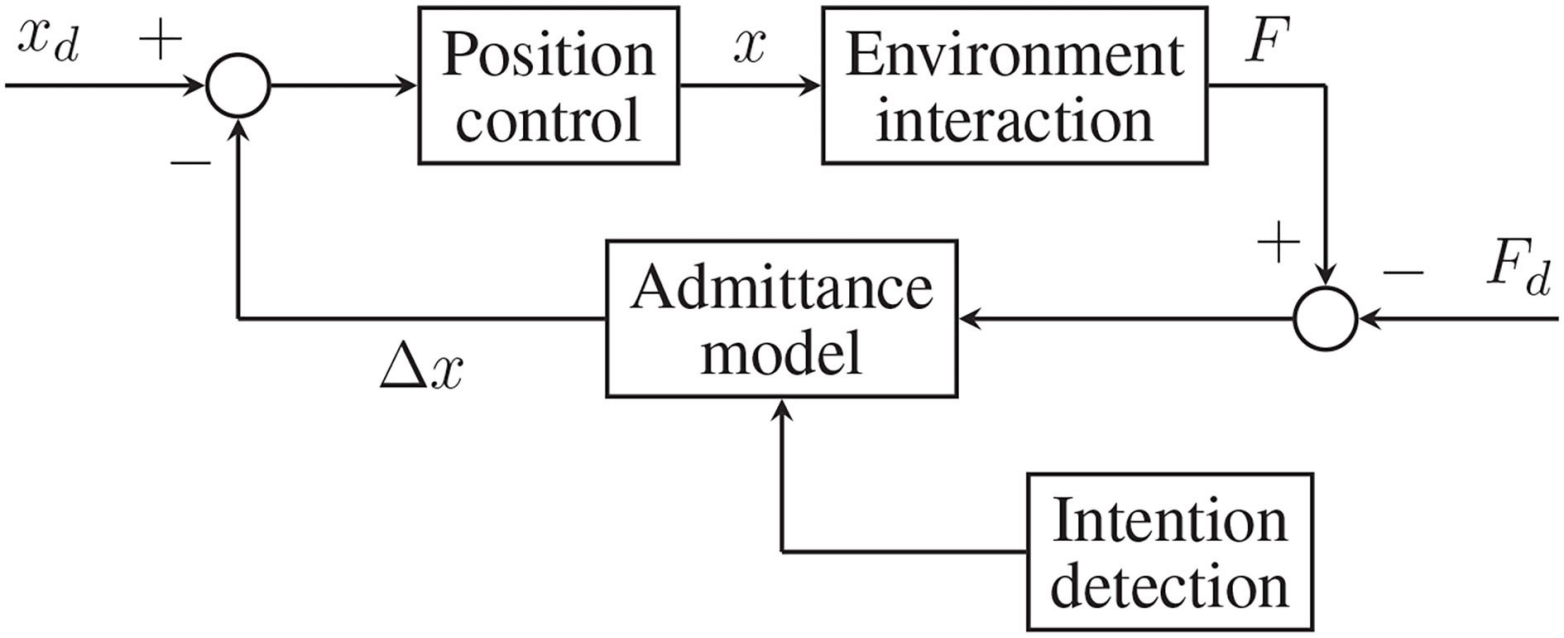
Variable admittance control scheme with force-tracking capabilities.

Variable admittance strategies (see Figure 7) are the ones that aim at imitating human's approach to motion: specifically, humans change their bodies, dynamic properties while performing complex movements like walking or interacting with different objects (Hogan, 1984). This same idea has been explored in the human-robot interaction field resulting in many different strategies (Ikeura et al., 1994; Tsumugiwa et al., 2002; Duchaine and Gosselin, 2007; Abu-Dakka and Saveriano, 2020). The user's intention is detected during a preliminary phase through sensors and inference algorithms, then input into the admittance model and proper parameters values (*K*, *B*, and *M*) are computed with some heuristics technique (Lee and Buss, 2008; Song et al., 2019). One of these methods has been implemented and tested in this project and will be described more in detail in section 3.2.

## 3. Methodology

In this section, the implementation details of the proposed overall architecture will be deeply highlighted. First, the several design guidelines upon which the virtual environment has been built and validated will be illustrated. Subsequently, the implemented admittance control strategies will be described by considering both the theoretical aspects as well as the experimental outcomes.

### 3.1. VR Environment Selection and Development

In order to design a suitable VR environment, several required features have been taken into accounts. Primarily, the physics simulator framework or library needs to provide the possibility to build custom robots and/or robot-like objects so as to straightforwardly implement a faithful digital replica of the BMIFOCUS exoskeleton device. In the second place, the virtual twin should be capable of being motion controlled while supplying realistic sensory feedback signals. Additionally, the digital exoskeleton requires to be embeddable in software architecture (e.g., the ROS framework) enabling an effortless, peer-to-peer interaction with the real BMIFOCUS device as far as both the exchanged force signals and a comprehensive visual representation are concerned. Motivated by these considerations, three distinct physics simulators have been identified as appropriate to fit the above-mentioned requirements: Gazebo, Pybullet, and Webots. A brief overview of each framework along with the respective features and drawbacks will be introduced to argue the final selection for this research activity.

**Gazebo** represents the default ROS physics simulator and, therefore, has been extensively employed for robotics simulations in a wide range of application fields. Based on the Bullet Physics library, the robots properties can be easily defined in the Universal Robot Description Format (URDF) format^6^, and the robot links can be driven by means of dedicated ROS packages. Despite being specifically integrated for a ROS-based software architecture, this solution has been discarded after several excessively unstable simulation tests caused by numerical approximations of the complex exoskeleton kinematics.

**Pybullet** is the python binding of the Bullet Physics library and is strongly recommended for robotics and VR applications. Its multi-thread internal structure allows for straightforward incorporation with ROS: a robot structure can be loaded from a URDF file whereas the simulation itself is handled by the library API. However, even in this case, the simulation of the BMIFOCUS exoskeleton closed chain kinematics has outlined not negligible undesired behaviors leading to an overall instability of the simulated scene.

Finally, **Webots: robot simulator** has been checked as well. Based on the Open Dynamics Engine (ODE) project, contrary to Gazebo and Pybullet, it does not make use of the URDF standard for the robot definition. Conversely, the VRML97 description language is exploited. A specific ROS package, *webots_ros*^7^, is provided so as to smoothly integrate the Webots API controllers into ROS nodes. Arising more stable than the others, Webots has been picked as the ideal solution to handle the VR requirements explained in the first paragraph of this section.

**Figure 8:**
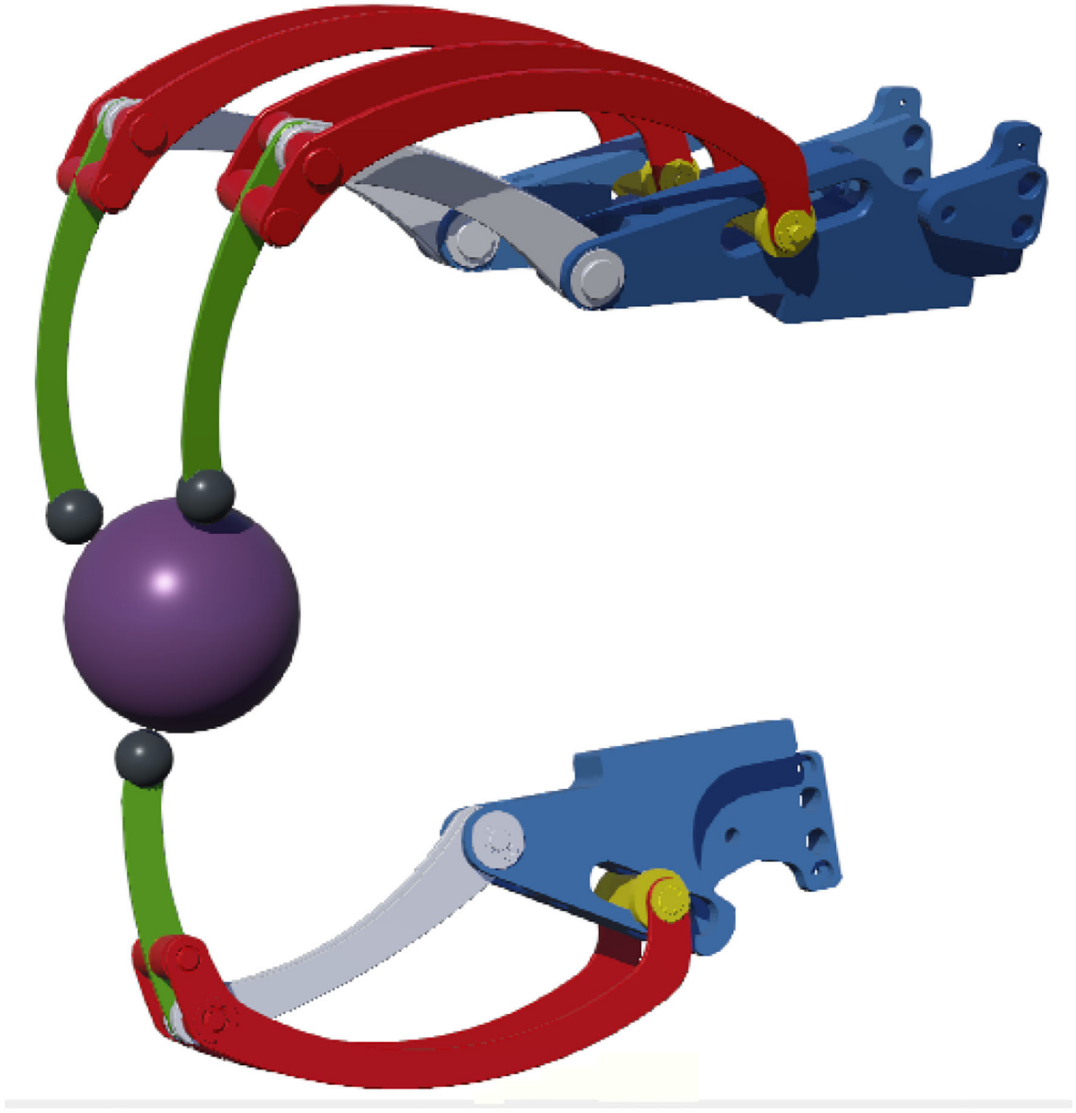
The Hand Exoskeleton System (HES) digital-twin developed within the Webots virtual environment. The purple sphere has been added to simulate interaction with objects.

**Figure 9:**
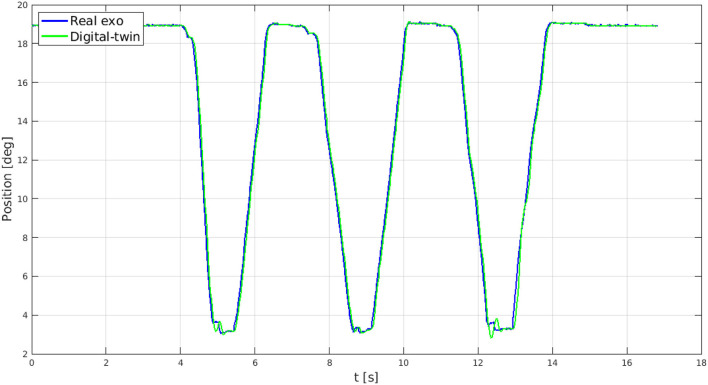
Comparison between the angular position of a real finger mechanism (in blue) and the one from the virtual replica (in green).

Once identified Webots as the proper virtual simulator, the BMIFOCUS exoskeleton digital-twin has been developed in order to provide visual guidance to a user during rehabilitation exercises as well as sensory feedback from the VR environment (as shown in Figure 8). The kinematics of the real device has been replicated by means of virtual components (i.e., links and joints) that physically mimic the mechanical characteristics of the real parts. Each virtual exoskeleton's finger mechanism is driven with a virtual motor positioned in correspondence with their only DOF so as to actuate them the same way the real ones are. These virtual actuators are position controlled with a PID following the real exoskeleton's position as a reference and regulated through a custom ROS node. Force feedback signals are measured from virtual force sensors placed on each virtual finger mechanism end-effector (the gray spheres in Figure 8). This solution enables a good matching of the virtual and real exoskeletons behavior (as shown in Figure 9) providing the perfect test bench to preliminarily try the proposed control strategy.

### 3.2. Proposed Admittance Control

**Figure 10:**
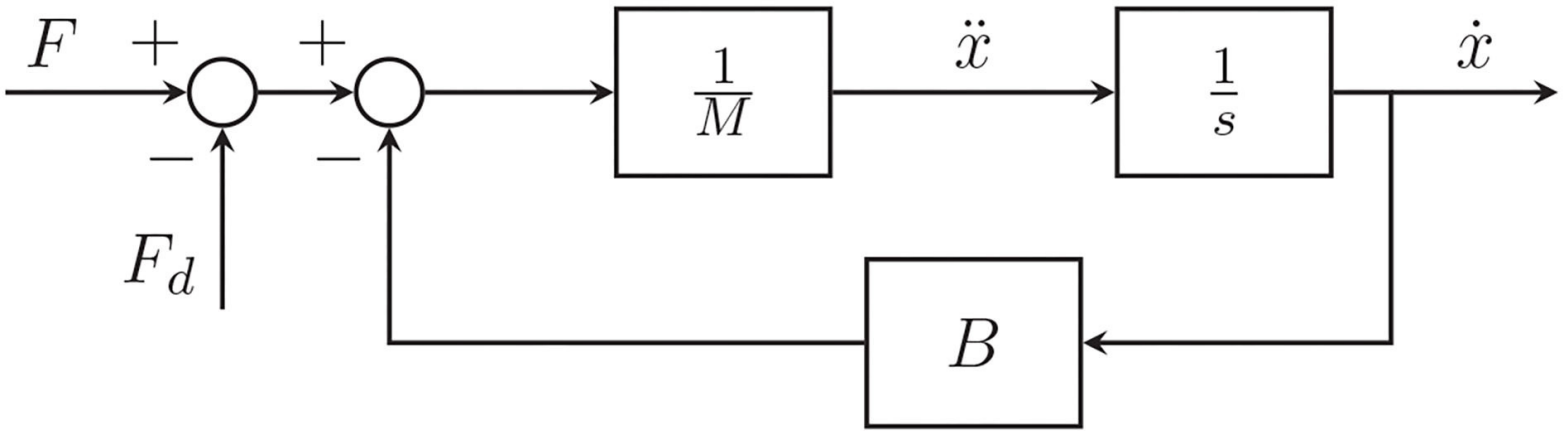
Block diagram of the admittance control strategy implemented.

As previously illustrated in section 2.3, an admittance control architecture comprises of two nested feedback loops: the outer regulates the desired dynamics and the inner correctly drives the actuation system. In this case, since the BMIFOCUS exoskeleton actuators are handled by a PID-based speed-controller, the admittance filter is designed so as to provide speed references for the exoskeleton device. More specifically, the proposed admittance control is decentralized over the three HES finger mechanism; therefore, despite being presented hereafter for a single finger approach, the implemented architecture has been extended to the whole set of mechanisms. Aiming not to impair the patient's movements and simulate a free motion when not handling virtual objects or, conversely, providing the patient with the correct force feedback when instead is interacting with them, the following admittance model has been adopted:


(5) $$\begin{array}{l} M\ddot{x}+B\dot{x}=F\left(t\right)-F_{d}\left(t\right) \end{array}$$

where *F*_*d*_(*t*) is supplied by the virtual force sensors. A block diagram representation is reported in Figure 10.

By further discretizing Equation 5 with sampling time *T*_*s*_, the speed reference at the discrete time step *k* for the PID control of the HES inner loop can be expressed as:


(6) $$\begin{array}{l} v\left(k\right)=v\left(k-1\right)+\frac{F\left(k\right)-F_{d}\left(k\right)-Bv\left(k-1\right)}{M}T_{s} \end{array}$$

Starting from this mathematical representation, two distinct admittance control strategies have been specifically designed, implemented, and tested:

- *Classical Admittance Control* (C-AC): the canonical controller with force-tracking ability reported in Equation 6;
- *Variable Admittance Control* (V-AC): the former C-AC controller was modified so as the inertia (*M*) and damping (*B*) terms are online adapted to the user's motion intention. As previously mentioned, such a procedure has been inspired by Lecours et al. (2012); however, the application field is clearly distinct from the original one. Furthermore, in Lecours et al. (2012), neither a desired reference force nor a VR system, source of such force reference value, were introduced.

Both the mentioned methodologies will be hereafter detailed along with a comparative analysis of the achieved results.

#### 3.2.1. Classical Admittance Control

As outlined in Equation 5, this technique requires the inertia as well as the damping term to be heuristically tuned. From a qualitative point of view, in order to have a fast dynamic response, very low inertia is desired; on the other hand, an excessively low value may cause system instability. Consequently, after a precise parameter-tuning stage, a final value *M* = 0.008 *kgm*^2^ has been selected, slightly larger than the estimated stability threshold of *M* = 0.005 *kgm*^2^ (refer to section 2.3.2). Turning to the damping tuning procedure, several different values have been tested. Tests highlighted that a large damping value provides a fast filter response characterized by a reactive reference modification following the trend of the force measurements from the HES. However, as reported in section 2.3.2, high damping values may also cause system instability. As a result, the value of *B* = 0.145 *Ns*/*m* has been set as the optimal damping value for the proposed system.

#### 3.2.2. Variable Admittance Control (V-AC)

As previously mentioned, this approach has been implemented relying on the key idea to real-time vary the admittance model parameters so as to actively assist the patient's desired motion and enhance the BMIFOCUS device transparency. Indeed, the inertia and damping coefficient of Equation 5 are adjusted online according to the user's intention. A primary heuristic criterion has been employed to achieve the user's motion detection; if the actual finger mechanism angular acceleration and velocity show the same direction (i.e., have the same sign), the intention to keep moving further is detected; otherwise, the desire to stop or invert the motion is inferred.

Once the user's intention is detected, the approach proposed in Lecours et al. (2012) is implemented to achieve the variable-admittance behavior. In the first case, in order to promote acceleration and thus the device responsivity, the desired damping *B* is decreased by exploiting a correction factor proportional to the desired acceleration;


(7) $$\begin{array}{l} B_{acc}=B_{f}-\alpha |{\ddot{x}}_{d}| \end{array}$$

where *B*_*f*_ is the apriori defined damping default value *B*_*f*_ = 0.145 *Ns*/*m*.

In the second case, by pursuing a coherent approach, the damping values are increased whenever the user requires a deceleration phase:


(8) $$\begin{array}{l} B_{dec}=B_{f}+\alpha |{\ddot{x}}_{d}| \end{array}$$

Two different equations can be exploited to modify the inertia value according to the modified damping coefficient:


(9) $$\begin{array}{l} M_{acc}=M_{f}\;\frac{B_{acc}}{B_{f}} \end{array}$$

(10) $$\begin{array}{l} M_{dec}=M_{f}\;\frac{B_{dec}}{B_{f}}\left(1-\beta \left(1-e^{-\;\gamma \left(B_{dec}-B\right)}\right)\right) \end{array}$$

where *M*_*f*_ is the previously tuned inertia default value $M_{f}=0.008\;kgm^{2}$, and α, β, and γ are correction factors heuristically tuned to finely adjust the exoskeleton behavior. While accelerating is safely handled with the proportional action of Equation 9, decelerating needs to be tackled more carefully to avoid possible discontinuities due to the inversion of motion. The exponential function reported in Equation 10 is exploited to handle such possibilities.

However, during some preliminary tests for the system under investigation, the variation of the M parameter was found to be low and directed toward the instability margin (*M* = 0.005 *kgm*^2^). For this reason, the choice to leave the value of the inertia term to its default value *M* = 0.008 *kgm*^2^ seemed the most reasonable.

**Figure 11:**
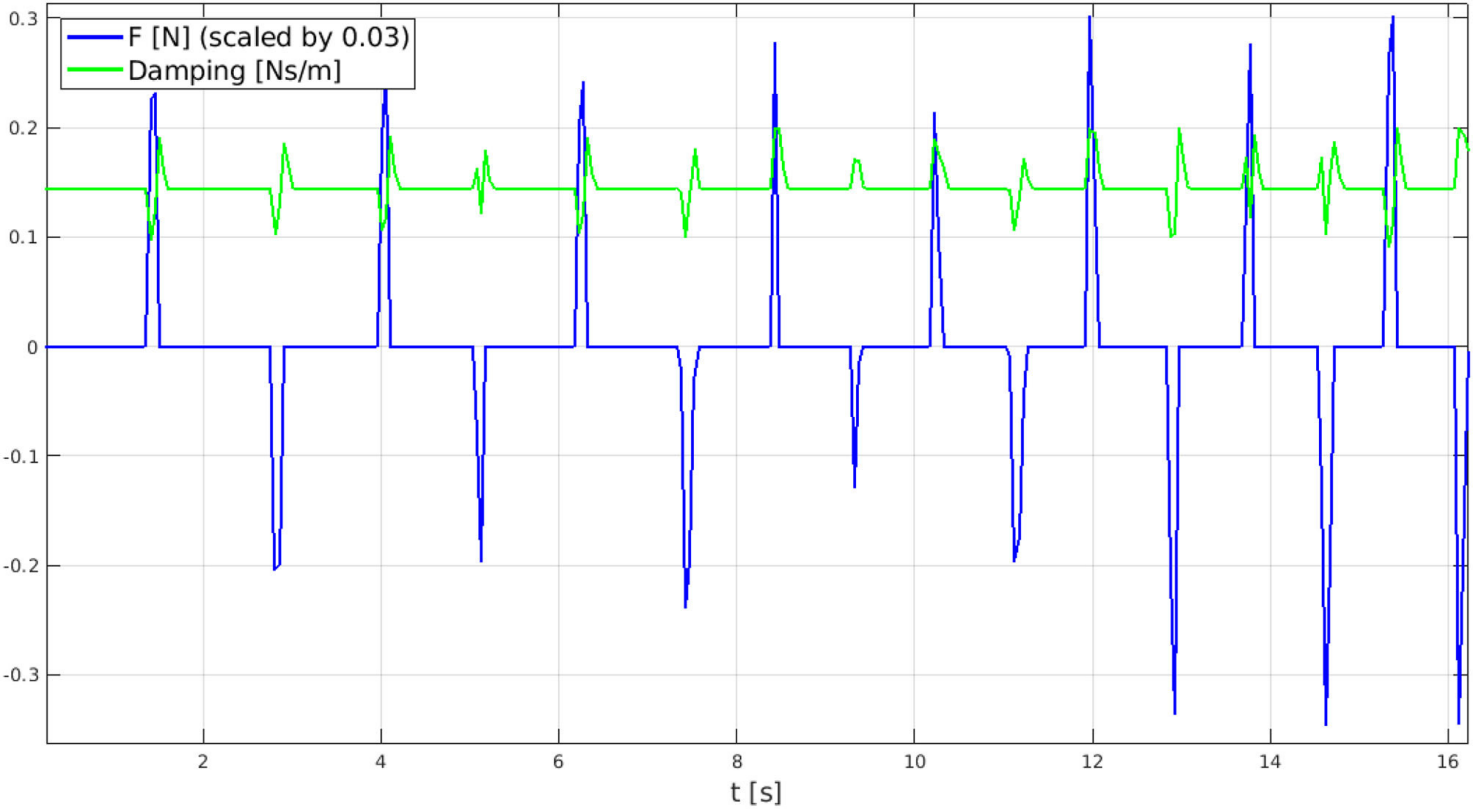
Real time damping variation (in green) according to force sensor measurements (in blue) with α = 0.0005 chosen after some tests. As reported within the legend, force measures are scaled with a 0.03 factor for plot readability.

It is primary to outline that as the inertia is kept constant while varying the damping value, the controller bandwidth, inferiorly limited by the damping to inertia ratio (*B*/*M*), would change as well. This effect may slow the system response during the acceleration phase and might cross the stability border when decelerating. In light of these observations, the damping value is constrained between 0.004 *Ns*/*m* and 0.2 *Ns*/*m* to preserve the system stability (*B* ≤ 0.25 *Ns*/*m*) and a minimum bandwidth of 0.5 *Hz*^8^ (*B*/*M* ≥ 0.5 *kgm*^2^). As a consequence of these design guidelines, the damping term does arise as the only independent parameter to be tuned for the V-AC approach and, for sake of brevity, just its variation along the detected user's intention will be reported. The variation of the damping coefficient following the user's interaction with the exoskeleton (namely, the user's intention) is reported in Figure 11.

As shown from the graph, the intention detection system performs as expected. The user exerts a force (either positive or negative) to accelerate, the damping parameter decreases to reduce its dissipating action; conversely, when the measured force drops back to zero, thus identifying a deceleration intention, the damping value increases to quickly stop the motion. As can be further expected from the V-AC mathematical formulation, for large α values, the damping increase is sharper. After some tests, a suitable value of α = 0.0005 has been identified.

## 4. Tests and Results

This section illustrates the overall interaction between the admittance controlled HES and the developed Webots-based VR environment. In order to evaluate the functionality of the whole system a pilot study composed of two distinct experiments has been setup: (i) in the first proposed scenario, the *free motion* mode has been considered; (ii) second, a trial with a graspable virtual object (namely, the sphere visible in Figure 8) has been arranged. The choice of these two experimental setups has been delineated to clearly outline both the transparency of the device and the capability to render force feedback. As already reported, both these characteristics are crucial when it comes to rehabilitative robot systems as the basis for the implementation of safe, customizable, engaging, and stimulating VR-based exercises for patients. The tests have been conducted involving a single healthy subject (male, 27 years old, trained to interact with the HES and the VR) as the exoskeleton geometry is optimized to fit a specific target hand (as reported in Bartalucci et al., 2020). Since the admittance control strategy is decentralized and replicated over each finger with a shared Webots VR, for the sake of simplicity, just the results of a single finger are reported.

### 4.1. Preliminary Assessment

In order to provide a preliminary objective analysis of the proposed AC methodologies, the system responses to custom, pre-recorded force signals have been studied. The need for such a study arises for both inspecting, in a uniform way, the strategy outputs for equivalent inputs as well as for a pragmatical necessity, since repeatedly providing an identical force signal multiple times does result as impossible even for an expert user. In particular, with the aim of achieving force signals as realistic as possible, instead of exploiting simulated, scripted, force signals, the subject has been asked to apply two distinct forces on the load cell: a single impulse as well as a variable-frequency impulse sequence.

**Figure 12:**
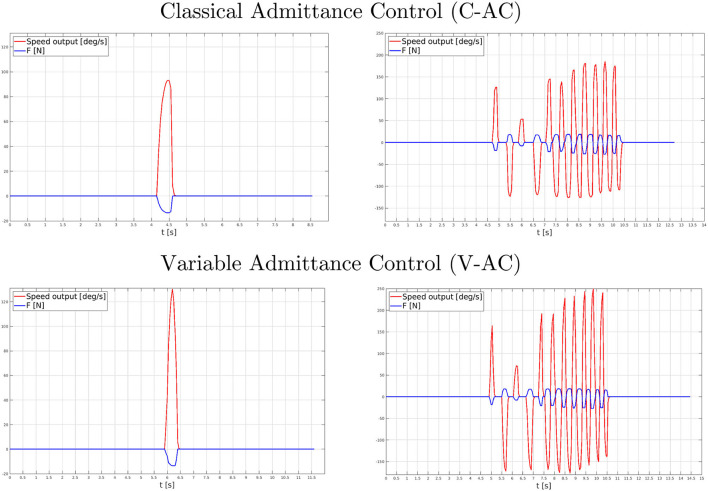
Performance comparison between the C-AC (top graph) and V-AC (bottom graph) during the preliminary assessment tests. On the left, the control response to a single force impulse; on the right, the control response to a variable-frequency impulse train.

Figure 12 shows the C-AC and V-AC methodology outcomes as long as both the two different force signals have been applied. In particular, a graphical investigation outlines the correct functionality of both the strategies (i.e., the reference speed values coherently react to the input force signals). Nevertheless, the promptness capability evidently differs between the two approaches: V-AC increases and decays the velocity output more rapidly, with larger amplitude variations, than C-AC by achieving a HES device more reactive to follow the user's intentions.

### 4.2. Free Motion Mode

The V-AC strategy arose to be the most reactive during the preliminary assessment. However, before claiming which of the two approaches was best suited for rehabilitation application, the actual results of such reactivity had to be tested in a real-use scenario, in which the actual interaction between the HES and the user had to be analyzed.

**Figure 13:**
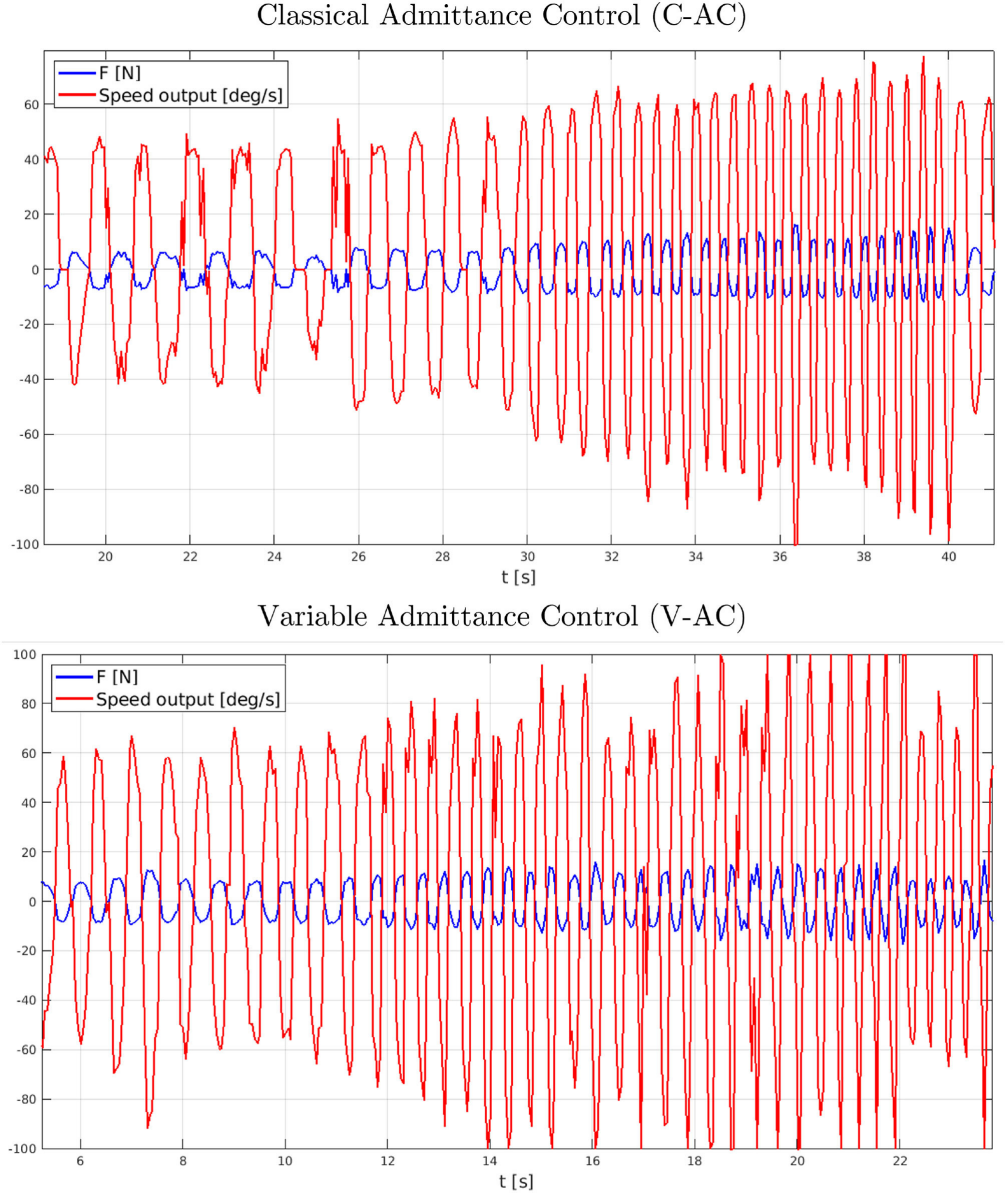
Performance comparison between the C-AC (top graph) and V-AC (bottom graph) during the free motion mode tests.

In order to compare the performance of the two proposed control strategies, data has been acquired while performing some repetitive motion of the index finger. More specifically, the index finger mechanism has been worn on a healthy subject and, then, the wearer has been asked to repeatedly tap the finger with variable frequency. The HES was switched on and the control was set on *free motion* mode to assess how the exoskeleton would follow the user's intentions: since no virtual objects are introduced in the Webots VR, the desired reference forces, provided by the virtual force sensors, was set to zero. Force measurements have been collected from the load cell and compared with the speed reference output from the admittance model, as reported in Figure 13. During the test, the subject could also rely on visual feedback coming from the VR following the exoskeleton motion.

The first designed experiment not only aimed to assess the BMIFOCUS HES transparency but also to identify the most suitable admittance control strategy for rehabilitation applications. These results have highlighted that both the C-AC and V-AC approaches show the ability to precisely follow the user's intentions, overall proving remarkable transparency of the BMIFOCUS exoskeleton. However, as clearly visible, the pHRI resulting from C-AC and V-AC was different when varying the tapping frequency. Before going into the details of the performance comparison, it is important to state that the results shown in Figure 13 represent the actual interaction between the user and the device: this implies that the interaction force (in blue) and the admittance model output (in red) mutually influence each other. In light of this premise, the graphs show that the pHRI produced by the C-AC method results in being overall noisy, while the V-AC one is smoother at low frequencies, while it appears noisier at high frequencies. In the first analysis, this behavior might derive from the higher reactivity of the V-AC strategy making this approach itself more prone to undesired patterns due to disturbances when the dynamics of the system increases. The evident noise reduction while performing pHRI with V-AC at low frequencies makes the interaction with the exoskeleton more natural and transparent. This feature, contextualized in a rehabilitation scenario results crucial, as the operating frequencies that characterize therapy sessions are usually low to guarantee patients' safety and comfort. In conclusion, although a quantitative evaluation comparison between the two implemented approaches outlines just minor differences, the V-AC technique has been selected as the control strategy that is most suitable for the application under investigation and has been hence exploited during the further tests about the interaction with virtual objects.

### 4.3. Interaction With Virtual Objects

The second trial focused instead on the interaction with virtual objects to be used to enhance the immersivity of rehabilitation exercises. This key property is achieved thanks to the force-tracking ability of the developed admittance control strategy; such a valuable resource is carried out upon the following hierarchical, but still concurrent, stages:

- while the user moves his hand when wearing the exoskeleton the digital-twin mirrors such motion;
- as long as the virtual model does not encounter any obstacle, the system persists in the free motion mode and the reference force tracked by the admittance control system has zero value;
- once the virtual HES comes into contact with a virtual object, the Webots physics simulator engine generates a reaction force provided by a measured force from a virtual force sensor;
- the virtual force values are used as reference signals and, combined with the force feedback from the real exoskeleton, input into the implemented admittance control strategy;
- the admittance control supplies the velocity references for the HES internal PID controllers that, by tracking them, allow the finger mechanism to exert a reaction force on the user's finger;
- at this phase, if the patient is able to perform an identical but opposite force on the finger mechanism an equilibrium position will be reached: the digital-twin holding (or pushing) a virtual object while the user feels the reaction forces as if the object were in his/her hand.

**Figure 14:**
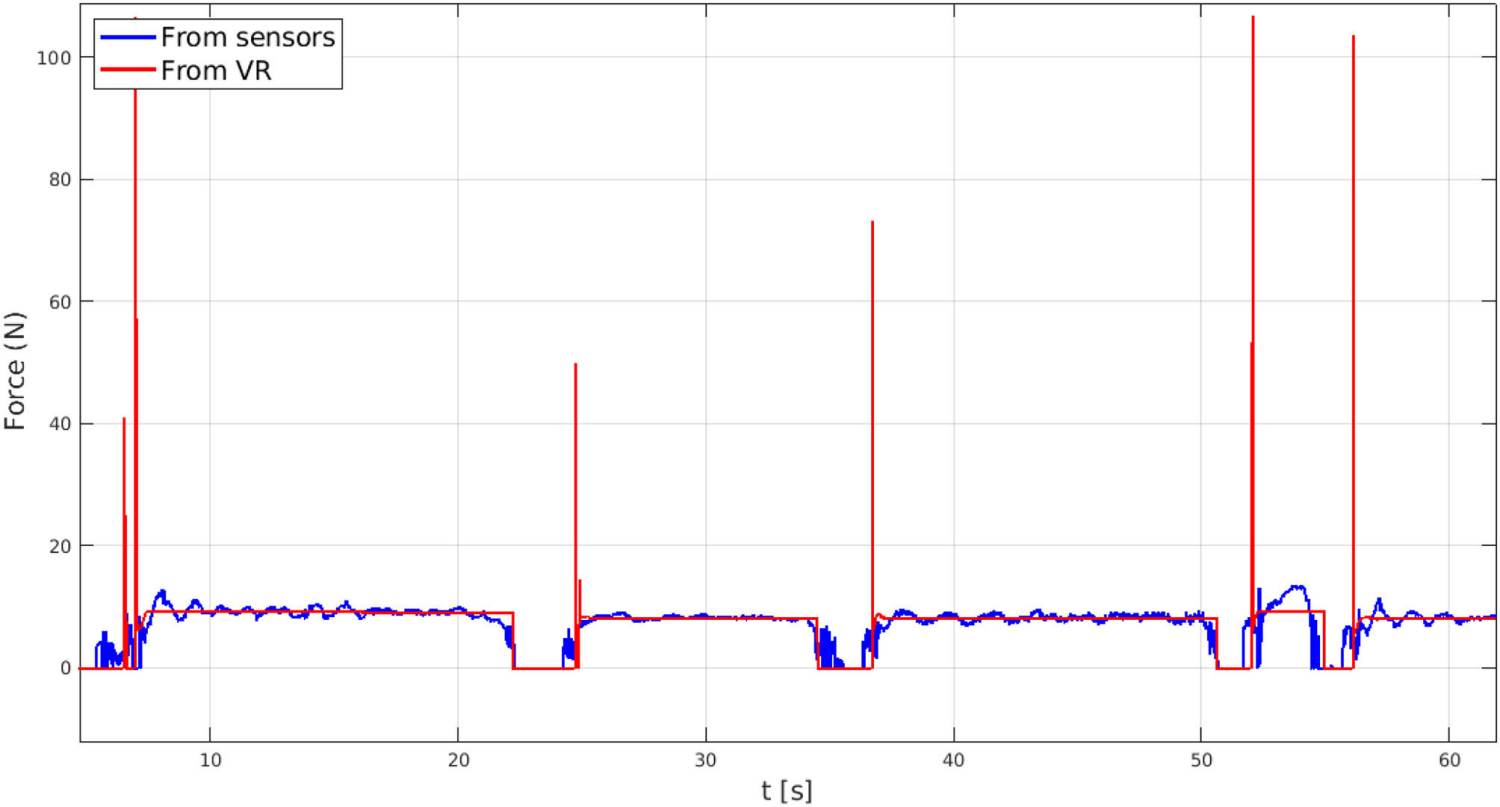
The force tracking performance of the implemented admittance control.

The results of the test with a virtual spherical object are reported hereafter. In Figure 14, the comparison between the force applied by the user on the exoskeleton and the one computed in VR is displayed. As the HES digital replica touches the virtual sphere in a grasping action, the simulated force sensor supplies a step-comparable force reference, which the admittance control architecture handles by accomplishing the force tracking feature. Instead, while the BMIFOCUS exoskeleton digital-twin is not in contact with the VR object, the desired reference force is set to zero and *free motion* mode is suitably fulfilled. For sake of completeness, it is worth noting that several steep peaks are present among the virtual reaction forces. This pattern, arising as impulsive forces due to the HES contact with a rigid object in the Webots framework, is, however, filtered by the mechanics of the system and does not cause excessive undesired motion and, therefore, is not perceived by the user.

## 5. Conclusion

The presented work describes the design process of a rehabilitation tool bringing together the benefits of robotic exoskeletons and VR. This research activity tackles a rehabilitation scenario in which a patient, suffering from hand reduced mobility, is requested to perform manipulation tasks. A pre-existing hand exoskeleton, designed and developed by the researchers at UNIFI DIEF in the framework of the regional research project BMIFOCUS, has been the basis of the presented research activity since it already exhaustively fulfilled the mechanical requirements for a safe, flexible, and comfortable robot-based rehabilitation. Due to the very nature of the device involved in this research activity, the treatment under consideration must necessarily take place in a clinic under the supervision of a therapist. The patient is asked to interact with virtual objects while wearing the exoskeleton. From this point of view, the integrated system (intended as a set of the exoskeleton and VR) has a 3-fold purpose: on the one hand, it provides help to the patient according to the guidelines of the AAN approach; on the other hand, it is a valuable tool for monitoring the progress of the patient in real-time by measuring both the kinematics of the hand and the forces exerted; finally, thanks to the interaction with VR, it can easily propose different exercises, adapted to the specific needs of the patient and designed in order to increase the patient's involvement in the exercises.

After a minute selection of a suitable physics simulator, a HES digital-twin has been realized in the *Webots: robot simulator* framework as well as thoroughly tested to check consistency with the real exoskeleton. Such a simulated device does represent a strategical tool in providing a patient with VR-based visual feedback over the therapy activity. Besides, it is proved that interactive VR environments integrated with physical devices arise as noteworthy tools to boost the patient's engagement during rehabilitation exercises by simulating the user immersively interacting with virtual scenes and objects. In particular, the proposed system simulates object grasping sensations, providing the patients with both force and visual feedback.

The scientific literature identifies admittance control as a satisfactory methodology to achieve the aforementioned purpose. Indeed, this procedure, along with its force-tracking ability, manifests the ability to simultaneously handle the end-effector position and contact force control. Besides, admittance control paves the way toward the shaping of the complex pHRI by allowing, with a proper tuning process of the inertia and damping terms, for a more transparent interaction between the user and the exoskeleton device. By exploiting this control technique, users can move their hands with a minimally invasive HES presence during free motion, while feeling opposing forces every time, a virtual grasping action is performed.

Two distinct architectures have been implemented and tested **involving a healthy subject**: classical (C-AC) and variable (V-AC) admittance control. C-AC reflects the original formulation with fixed inertia and damping values. V-AC varies the model parameters according to the patient's intention in order to better assist his desired movements. In this view, default damping values have been tuned by employing a trial and error process, while the user's motion detector, based on a heuristic criterion based on desired speed and acceleration, has been proven effective.

Finally, the interaction between the user, the HES, and the VR environment has been analyzed. Both the C-AC and V-AC control strategies have highlighted promising performance while following the user's free motion, proving HES remarkably transparent for the user. However, when it comes to rehabilitation applications, the V-AC resulted best suited thanks to the smoother behavior at low operating frequencies. Besides, in terms of force feedback rendering, the chosen strategy showed the potential to provide a noteworthy physical human-robot interaction.

In conclusion, the control strategy described in this pilot study presents an excellent starting point for the development of a complete rehabilitation system as described above. The further steps in the development of this device will certainly involve extending the sample of subjects involved in the tests, possibly including real patients and characterizing the actual forces of interaction with objects that the exoskeleton is capable of rendering.

## Data Availability Statement

The original contributions presented in the study are included in the article/supplementary material, further inquiries can be directed to the corresponding author.

## Ethics Statement

Ethical review and approval was not required for the study on human participants in accordance with the local legislation and institutional requirements. The patients/participants provided their written informed consent to participate in this study.

## Author Contributions

BA and AR have supervised the research activity. All authors contributed to the article and approved the submitted version.

## Funding

This work has been supported by the Don Carlo Gnocchi Foundation and by two Italian projects: the HERMES project, funded by the Ente Cassa di Risparmio di Firenze, and the BMIFOCUS, funded by the Tuscany Region (POR FESR 2014–2020).

## Conflict of Interest

The authors declare that the research was conducted in the absence of any commercial or financial relationships that could be construed as a potential conflict of interest.

## Publisher's Note

All claims expressed in this article are solely those of the authors and do not necessarily represent those of their affiliated organizations, or those of the publisher, the editors and the reviewers. Any product that may be evaluated in this article, or claim that may be made by its manufacturer, is not guaranteed or endorsed by the publisher.

## Footnotes

^1^http://gazebosim.org (last accessed: 28th September 2021).

^2^https://pybullet.org (last accessed: 28th September 2021).

^3^https://cyberbotics.com (last accessed: 28th September 2021).

^4^https://www.ros.org (last accessed: 28th September 2021).

^5^According to the HES architecture presented in Bartalucci et al. (2020), the finger mechanism end-effector acts on the corresponding finger middle phalanx.

^6^ http://wiki.ros.org/urdf

^7^ http://wiki.ros.org/webots_ros

^8^Such bandwidth of 0.5 *Hz* is intended as the lowest frequency the system has to operate and it has been identified (with the help of clinical staff) considering a worst-case scenario where the finger mechanisms are asked to fully open or close the corresponding finger at least every 2 seconds.

## References

[B1] Abu-Dakka F. J. Saveriano M. (2020). Variable impedance control and learning-a review. arXiv preprint arXiv:2010.06246. 10.3389/frobt.2020.590681 33501348 PMC7805898

[B2] Anam K. Al-Jumaily A. A. (2012). Active exoskeleton control systems: State of the art. Procedia Eng. 41:988–994. 10.1016/j.proeng.2012.07.27317271386

[B3] Bartalucci L. Secciani N. Gelli J. Della Valle A. Ridolfi A. Allotta B. (2020). “Rehabilitative hand exoskeleton system: a new modular mechanical design for a remote actuated device,” in The International Conference of IFToMM ITALY (Naples: Springer), 128–136.

[B4] Bielsa V. F. (2021). Virtual reality simulation in plastic surgery training. literature review. J. Plastic Reconstr. Aesthet. Surg. 74, 2372–2378. 10.1016/j.bjps.2021.03.066 33972199

[B5] Colgate J. E. Hogan N. (1988). Robust control of dynamically interacting systems. Int. J. Control. 48, 65–88. 10.1080/00207178808906161

[B6] Conti R. Meli E. Ridolfi A. Bianchi M. Governi L. Volpe Y.. (2017). Kinematic synthesis and testing of a new portable hand exoskeleton. Meccanica 52, 2873–2897. 10.1007/s11012-016-0602-0

[B7] de Araújo A. V. L. Neiva J. F. d. O Monteiro C. B. d. M. Magalh aes F. H. (2019). Efficacy of virtual reality rehabilitation after spinal cord injury: a systematic review. Biomed. Res. Int. 2019:7106951. 10.1155/2019/7106951 31828120 PMC6885151

[B8] du Plessis T. Djouani K. Oosthuizen C. (2021). A review of active hand exoskeletons for rehabilitation and assistance. Robotics 10, 40. 10.3390/robotics10010040

[B9] Duchaine V. Gosselin C. M. (2007). “General model of human-robot cooperation using a novel velocity based variable impedance control,” in Second Joint EuroHaptics Conference and Symposium on Haptic Interfaces for Virtual Environment and Teleoperator Systems (WHC'07) (Tsukuba: IEEE), 446–451.

[B10] Gomi H. Kawato M. (1993). Neural network control for a closed-loop system using feedback-error-learning. Neural Netw. 6, 933–946. 10.1016/S0893-6080(09)80004-X

[B11] Hogan N. (1984). Adaptive control of mechanical impedance by coactivation of antagonist muscles. IEEE Trans. Automat. Contr. 29, 681–690. 10.1109/TAC.1984.110364425655955

[B12] Hogan N. Buerger S. P. (2018). “Impedance and interaction control,” in Robotics and Automation Handbook (Boca Raton, FL: CRC Press), 375–398.

[B13] Hua J. Zeng L. Li G. Ju Z. (2021). Learning for a robot: Deep reinforcement learning, imitation learning, transfer learning. Sensors 21, 1278. 10.3390/s21041278 33670109 PMC7916895

[B14] Huang G. Zhang W. Meng F. Yu Z. Chen X. Ceccarelli M.. (2018). Master-slave control of an intention-actuated exoskeletal robot for locomotion and lower extremity rehabilitation. Int. J. Precision Eng. Manufact. 19, 983–991. 10.1007/s12541-018-0116-x

[B15] Huang R. Cheng H. Qiu J. Zhang J. (2019). Learning physical human-robot interaction with coupled cooperative primitives for a lower exoskeleton. IEEE Trans. Autom. Sci. Eng. 16, 1566–1574. 10.1109/TASE.2018.288637627295638

[B16] Ikeura R. Monden H. Inooka H. (1994). “Cooperative motion control of a robot and a human,” in Proceedings of 1994 3rd IEEE International Workshop on Robot and Human Communication (Nagoya: IEEE), 112–117.

[B17] Ikeura R. Moriguchi T. Mizutani K. (2002). “Optimal variable impedance control for a robot and its application to lifting an object with a human,” in Proceedings. 11th IEEE International Workshop on Robot and Human Interactive Communication (Berlin: IEEE), 500–505.

[B18] Jung S. Hsia T. (1998). Neural network impedance force control of robot manipulator. IEEE Trans. Ind. Electr. 45, 451–461. 10.1109/41.67900327295638

[B19] Jung S. Hsia T. C. Bonitz R. G. (2004). Force tracking impedance control of robot manipulators under unknown environment. IEEE Trans. Control Syst. Technol. 12, 474–483. 10.1109/TCST.2004.82432027295638

[B20] Kavanagh S. Luxton-Reilly A. Wuensche B. Plimmer B. (2017). A systematic review of virtual reality in education. Themes Sci. Technol. Educ. 10, 85–119.

[B21] Keemink A. Q. van der Kooij H. Stienen A. H. (2018). Admittance control for physical human-robot interaction. Int. J. Rob. Res. 37, 1421–1444. 10.1177/027836491876895033826517

[B22] Kim H. Kwon J. Oh Y. You B. J. Yang W. (2018). “Weighted hybrid admittance-impedance control with human intention based stiffness estimation for human-robot interaction,” in 2018 IEEE/RSJ International Conference on Intelligent Robots and Systems (IROS) (Madrid: IEEE), 1–6.

[B23] Lecours A. Mayer-St-Onge B. Gosselin C. (2012). “Variable admittance control of a four-degree-of-freedom intelligent assist device,” in 2012 IEEE International Conference on Robotics and Automation (Saint Paul, MN: IEEE), 3903–3908.

[B24] Lee K. Buss M. (2008). Force tracking impedance control with variable target stiffness. IFAC Proc. 41, 6751–6756. 10.3182/20080706-5-KR-1001.01144

[B25] Lei C. Sunzi K. Dai F. Liu X. Wang Y. Zhang B.. (2019). Effects of virtual reality rehabilitation training on gait and balance in patients with parkinson's disease: A systematic review. PLoS ONE 14:e0224819. 10.1371/journal.pone.0224819 31697777 PMC6837756

[B26] Li Z. Liu J. Huang Z. Peng Y. Pu H. Ding L. (2017). Adaptive impedance control of human-robot cooperation using reinforcement learning. IEEE Trans. Ind. Electron. 64, 8013–8022. 10.1109/TIE.2017.269439127295638

[B27] Losey D. P. McDonald C. G. Battaglia E. O'Malley M. K. (2018). A review of intent detection, arbitration, and communication aspects of shared control for physical human-robot interaction. Appl. Mech. Rev. 70, 010804. 10.1115/1.4039145

[B28] Lu Z. Goldenberg A. A. (1995). Robust impedance control and force regulation: Theory and experiments. Int. J. Rob. Res. 14, 225–254. 10.1177/027836499501400303

[B29] Lum P. S. Burgar C. G. Shor P. C. Majmundar M. Van der Loos M. (2002). Robot-assisted movement training compared with conventional therapy techniques for the rehabilitation of upper-limb motor function after stroke. Arch. Phys. Med. Rehabil. 83, 952–959. 10.1053/apmr.2001.33101 12098155

[B30] Molteni F. Gasperini G. Cannaviello G. Guanziroli E. (2018). Exoskeleton and end-effector robots for upper and lower limbs rehabilitation: narrative review. PM R 10, S174–S188. 10.1016/j.pmrj.2018.06.005 30269804

[B31] Petrenko V. Tebueva F. Gurchinsky M. Ryabtsev S. Trofimuk O. (2019). “Exoskeleton for operator's motion capture with master-slave control,” in 7th Scientific Conference on Information Technologies for Intelligent Decision Making Support (ITIDS 2019) (Ufa: Atlantis Press), 152–158.

[B32] Pfandler M. Lazarovici M. Stefan P. Wucherer P. Weigl M. (2017). Virtual reality-based simulators for spine surgery: a systematic review. Spine J. 17, 1352–1363. 10.1016/j.spinee.2017.05.016 28571789

[B33] Qian C. Li W. Jia T. Li C. Lin P.-J. Yang Y.. (2021). Quantitative assessment of motor function by an end-effector upper limb rehabilitation robot based on admittance control. Appl. Sci. 11, 6854. 10.3390/app11156854

[B34] Radianti J. Majchrzak T. A. Fromm J. Wohlgenannt I. (2020). A systematic review of immersive virtual reality applications for higher education: design elements, lessons learned, and research agenda. Comput. Educ. 147:103778. 10.1016/j.compedu.2019.103778

[B35] Rose T. Nam C. S. Chen K. B. (2018). Immersion of virtual reality for rehabilitation-review. Appl. Ergon. 69, 153–161. 10.1016/j.apergo.2018.01.009 29477323

[B36] Roveda L. Iannacci N. Vicentini F. Pedrocchi N. Braghin F. Tosatti L. M. (2015). Optimal impedance force-tracking control design with impact formulation for interaction tasks. IEEE Rob. Autom. Lett. 1, 130–136. 10.1109/LRA.2015.250806127295638

[B37] Sado F. Sidek S. N. Yusof H. M. (2014). “Adaptive hybrid impedance control for a 3dof upper limb rehabilitation robot using hybrid automata,”. in 2014 IEEE Conference on Biomedical Engineering and Sciences (IECBES) (Kuala Lumpur: IEEE), 596–601.

[B38] Sandison M. Phan K. Casas R. Nguyen L. Lum M. Pergami-Peries M.. (2020). “HandMATE: wearable robotic hand exoskeleton and integrated android app for at home stroke rehabilitation,” in 2020 42nd Annual International Conference of the IEEE Engineering in Medicine Biology Society (EMBC) (Montreal, QC: IEEE), 4867–4872. 10.1109/EMBC44109.2020.9175332 PMC8485422 33019080

[B39] Schumacher M. Wojtusch J. Beckerle P. von Stryk O. (2019). An introductory review of active compliant control. Rob. Auton. Syst. 119, 185–200. 10.1016/j.robot.2019.06.00923453170

[B40] Seraji H. Colbaugh R. (1997). Force tracking in impedance control. Int. J. Rob. Res. 16, 97–117. 10.1177/027836499701600107

[B41] Shi D. Zhang W. Zhang W. Ding X. (2019). A review on lower limb rehabilitation exoskeleton robots. Chin. J. Mech. Eng. 32, 1–11. 10.1186/s10033-019-0389-8

[B42] Song P. Yu Y. Zhang X. (2019). A tutorial survey and comparison of impedance control on robotic manipulation. Robotica 37, 801–836. 10.1017/S026357471800133930886898

[B43] Souzanchi-K. M. Arab A. Akbarzadeh-T M.-R. Fateh M. M. (2017). Robust impedance control of uncertain mobile manipulators using time-delay compensation. IEEE Trans. Control Syst. Technol. 26, 1942–1953. 10.1109/TCST.2017.273910927295638

[B44] Staubli P. Nef T. Klamroth-Marganska V. Riener R. (2009). Effects of intensive arm training with the rehabilitation robot armin ii in chronic stroke patients: four single-cases. J. Neuroeng. Rehabil. 6, 1–10. 10.1186/1743-0003-6-46 20017939 PMC2807864

[B45] Tsumugiwa T. Yokogawa R. Hara K. (2002). “Variable impedance control based on estimation of human arm stiffness for human-robot cooperative calligraphic task,” in Proceedings 2002 IEEE International Conference on Robotics and Automation (Cat. No. 02CH37292), Vol. 1 (Washington, DC: IEEE), 644–650.

[B46] Wang P. Wu P. Wang J. Chi H.-L. Wang X. (2018). A critical review of the use of virtual reality in construction engineering education and training. Int. J. Environ. Res. Public Health 15, 1204. 10.3390/ijerph15061204 29890627 PMC6025066

[B47] Wolfartsberger J. (2019). Analyzing the potential of virtual reality for engineering design review. Autom. Construct. 104, 27–37. 10.1016/j.autcon.2019.03.018

[B48] Yung R. Khoo-Lattimore C. (2019). New realities: a systematic literature review on virtual reality and augmented reality in tourism research. Curr. Issues Tourism 22, 2056–2081. 10.1080/13683500.2017.1417359

